# The Emerging Role of Exosomes in Oral Squamous Cell Carcinoma

**DOI:** 10.3389/fcell.2021.628103

**Published:** 2021-02-22

**Authors:** Yanhui Lu, Zhichao Zheng, Yunyi Yuan, Janak L. Pathak, Xuechao Yang, Lijing Wang, Zhitong Ye, William C. Cho, Mingtao Zeng, Lihong Wu

**Affiliations:** ^1^Affiliated Stomatology Hospital of Guangzhou Medical University, Guangzhou Key Laboratory of Basic and Applied Research of Oral Regenerative Medicine, Guangzhou, China; ^2^Guangzhou Medical University School and Hospital of Stomatology, Guangzhou, China; ^3^Department of Orthodontics, Affiliated Stomatology Hospital of Guangzhou Medical University, Guangzhou Key Laboratory of Basic and Applied Research of Oral Regenerative Medicine, Guangzhou, China; ^4^Vascular Biology Research Institute, Guangzhou Higher Education Mega Center, Guangdong Pharmaceutical University, Guangzhou, China; ^5^Department of Clinical Oncology, Queen Elizabeth Hospital, Kowloon, Hong Kong; ^6^Center of Emphasis in Infectious Diseases, Department of Molecular and Translational Medicine, Paul L. Foster School of Medicine, Texas Tech University Health Sciences Center El Paso, El Paso, TX, United States

**Keywords:** oral squamous cell carcinoma, non-coding RNAs, exosomes, tumor microenvironment, extracellular vesicles

## Abstract

Oral cancer constitutes approximately 2% of all cancers, while the most common type, oral squamous cell carcinoma (OSCC) represents 90% of oral cancers. Although the treatment of OSCC has improved recently, it still has a high rate of local recurrence and poor prognosis, with a 5-year survival rate of only 50%. Advanced stage OSCC tends to metastasize to lymph nodes. Thus, exploring new therapeutic strategies for OSCC is therefore an urgent priority. Exosomes, the small membrane vesicles derived from endosomes, have been detected in a wide array of bodily fluids. Exosomes contain a diversity of proteins, mRNAs, and non-coding RNAs, including microRNAs, long non-coding RNAs, piRNAs, circular RNAs, tsRNAs, and ribosomal RNAs, which are delivered to neighboring cells or even transported to distant sites. Exosomes have been associated with the tumorigenesis of OSCC, promote the proliferation, colonization, and metastasis of OSCC by transferring their contents to the target cells. Furthermore, exosomes are involved in the regulation of the tumor microenvironment to transform conditions favoring cancer progression *in vivo*. In this review, we summarize the crucial role of exosomes in the tumorigenesis and progression of OSCC and discuss the potential clinical application of exosomes in OSCC treatment.

## Introduction

Oral squamous cell carcinoma is the most common subtype of oral carcinoma, a genetic and epigenetic disease characterized by histopathological differentiation and a propensity for LNM (Bu et al., 2015). Surgical removal of tumors as well as pre- or post-operative chemotherapy, radiotherapy, and adjuvant therapies are the main strategies to increase survival (Coyte et al., 2014; Adel et al., 2016). Although treatment outcomes for OSCC have been improved recently, the prognosis for OSCC is still poor, the 5-year survival rate is reported as 50%, due to late diagnosis which resulting infeasibility of curative resections. Furthermore, the prognosis for this disease is poor due to metastatic invasion, with a propensity for local recurrence and distant metastasis (Huang et al., 2019). OSCC dissemination targets both local tissues via direct invasion and distant sites by seeding pre-metastatic niches through secreted elements, including exosomes (Qiu et al., 2019). Considering the risk of late diagnosis of OSCC, improvements in prevention, early diagnosis, and treatment efficacy are urgently needed (Csõsz et al., 2017).

**FIGURE 1:**
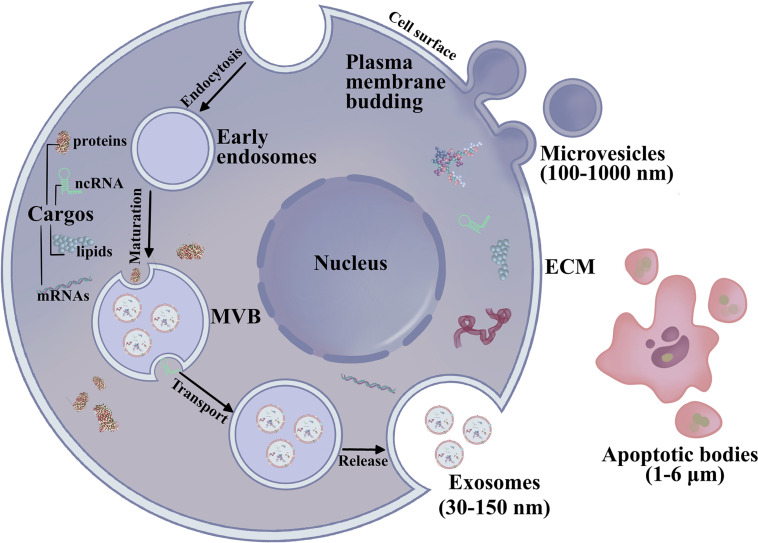
The biogenesis and secretion of EVs. Exosomes (30–150 nm) are generated by membrane endocytosis. Microvesicles (100–1,000 nm) are formed by the plasma membrane budding. Microvesicles are irregular in shape and heterogeneous in population. The largest EVs are apoptotic bodies (1–6 μm) are involved in the pathological condition.

According to the International Society for Extracellular Vesicles (ISEV), EVs are nano-size lipid bilayer vesicles released naturally from the cells to the ECM (Kalra et al., 2012; Thery et al., 2018; Jabbari et al., 2020b) (Figure 1). Generally, EVs are categorized as exosomes, microvesicles, and apoptotic bodies (Ahmadi and Rezaie, 2020; Jabbari et al., 2020a). Exosomes are small, membranous, extracellular microvesicles (∼30–150 nm in diameter) of endocytic origin. The formation of exosomes includes the beginning, endocytosis, MVB creation, and finally exosome secretion (Mellman, 1996; Keller et al., 2006; Raposo and Stoorvogel, 2013; Abak et al., 2018). Previous studies showed that non-coding RNAs (ncRNAs), mRNAs, proteins, and DNA fragments can be carried as “cargo” in EVs, which could serve as novel diagnostic biomarkers for OSCC (Raposo and Stoorvogel, 2013; Abak et al., 2018; Gai et al., 2018; Pourhanifeh et al., 2020). Another study found that exosomes can reprogram signal transduction under pathophysiological conditions and deliver important proteins as mediators (Li et al., 2019d; Amiri et al., 2021). These studies demonstrated that exosomes are present in the saliva of healthy donors, and exosomes have been reported in other body fluids, such as blood, cerebrospinal fluid, serous cavity effusion, and urine (Cao et al., 2020). Exosomes play an essential role in mediating signal transport for intercellular communication or over long distances by transporting microRNA (miRNAs), mRNAs, and proteins (Ohno et al., 2013a; Zhang et al., 2015; Sadri Nahand et al., 2020). Exosomal miRNAs are potential diagnostic biomarkers for various malignancies, regulating protein expression in cell proliferation, tumor metastasis, cell apoptosis, genomic instability, and immune responses. Wang H. et al. (2014) showed that exosomal miR-21 in the serum of OSCC patients is higher than that in chronic hepatitis patients and healthy individuals, suggesting that serum miRNAs may act as diagnostic biomarkers for OSCC. Sohn et al. (2015) detected serum exosomal miRNAs in patients with CHB, liver cirrhosis, and OSCC with CHB and discovered that a panel of eight exosomal miRNAs are significantly different between the OSCC and chronic hepatitis or liver cirrhosis groups. These efforts have revealed that exosomal contents can be transported across biological membranes and that exosomes have a role in OSCC development. Exosomes and their pattern of transfer deserve more attention to clearly define their roles in OSCC diagnosis and therapeutics. This review highlights exosomes in exchanges between normal and OSCC cells as well as the endocrine transport of exosomes from distant cells via the TME. We also review recent advances in exosomes in cancer initiation, progression and their potential clinical relevance to OSCC.

## Exosome Biogenesis, Isolation Methods, and Biological Functions

### The Biogenesis of Exosomes

The intraluminal vesicles (ILVs) are formed through inside budding in the multivesicular bodies (MVB) during the maturation of early endosomes to late endosomes which is regulated by ceramide (Nahand et al., 2020a). The cargos are encapsulated into ILVs during budding. However, the mechanism controlling cargo sorting is rather complicated and still unclear (Leidal and Debnath, 2020). EVs have overlapping sizes, similar morphology, and unspecific contents, resulting in difficulties in the isolation of specific subpopulations (Mathieu et al., 2019). The ILVs fuse with the cell membrane and are released into the extracellular space (Nahand et al., 2020b). It has been demonstrated that endosomal-tethering complexes necessitated for transport (ESCRT)-dependent and independent activities play an essential role in MVB formation (Hashemian et al., 2020). The ESCRT members including ESCRT-0, -I, -II, -III, and the related AAA-ATPase Vps4 complex recognize ubiquitinated membrane proteins leading to their internalization within the multivesicular endosome (Asgarpour et al., 2020; Ghaemmaghami et al., 2020). The MVB trafficking and secretion of exosomes are regulated by several members including the GTPase family (Rab11, Rab27a, Rab27b, and Rab35), heparanase, soluble NSF attachment receptor, and cytoskeleton regulatory proteins (Pant et al., 2012; Azmi et al., 2013; Beach et al., 2014; Abak et al., 2018). Furthermore, the dissemination process of the exosomes requires cellular stress, such as oxidative stress, hypoxia, etc (Abak et al., 2018). The released exosomes could transport into the recipient cells by the interaction between receptor-ligand, membrane fusion, and endocytosis through phagocytosis (Abak et al., 2018).

### Exosome Isolation Methods

The isolation methods of exosomes mainly include ultracentrifugation, size-based isolation, polymer precipitation, immunoaffinity, and microfluidic separation (Oeyen et al., 2018). Currently, ultracentrifugation is the most commonly used and the gold standard for exosome isolation. The advantage of ultracentrifuge is to treat lots of samples at one time. However, the problems of purity and damage the integrity of exosomes are the main disadvantages of the ultracentrifugation method (Tschuschke et al., 2020). PEG polymer combines the water molecules of the exosomes and reduces the solubility. PEG-based exosome isolation can be done in low-speed centrifugation with high efficiency (Doyle and Wang, 2019). The failure of the selection of exosomes and other EVs is the main drawback of PEG precipitation. Size-based exosome isolation is based on ultrafiltration and size exclusion chromatography. Ultrafiltration membrane isolates the exosomes by cutoff molecular sizes, which may lead to the exosome damage due to the pressure (Li et al., 2017). The size exclusion chromatography can be used to slow down the movement of small molecular, that protect the integrity and activity of exosomes. Magnetic bead and enzyme-linked immunosorbent assay are immunoaffinity-based exosome isolation methods, These methods capture the exosomes according to the antigen recognition of specific antibody (Zhang et al., 2020). Immunoaffinity-based exosome isolation generates pure exosomes with low yield. Microfluidic technology isolates exosomes in a short time with high purity, it differentiates physical and biochemical characteristics of exosomes by phosphatidylserine-specific proteins on exosomes (Jiang et al., 2020).

### The Biological Function of Exosomes

Under physiological conditions, exosomes are important mediators of cell-cell and inter-tissue communication. Exosomes exhibit important functions in regulating cellular activities during physiological and pathological conditions. During the cancer progression, different cells such as cancer cells, immune cells in TME generate exosomes that can transfer nucleotides and proteins among cells and participate in the complex pathogenesis of tumor development and metastasis (Ridder et al., 2014; Nazimek et al., 2016). Increasing evidence indicates that tumor cells communicate both with other tumor cells and with normal cells present in the TME via secretion and transfer of exosomal contents. Exosomal contents regulate tumor growth, angiogenesis, metastasis, sensitivity to chemotherapy, and immune evasion. Thus, it is essential to explore the effects of exosomes on OSCC development *in vitro* and *in vivo*.

## Exosomal miRNAs Are Essential for OSCC Development

### miRNAs in Exosomes

Among the bioactive components of exosomes, miRNAs can epigenetically alter gene function in the recipient cell, thus exerting their essential regulatory function on gene expression (Wang Y. et al., 2014). miRNAs are short ncRNAs of approximately 19–24 nucleotides in length (Chen et al., 2012b) and function to suppress the expression of protein-coding genes at the post-transcriptional level by degrading or inhibiting the translation of mRNAs (Bartel, 2004; Miska, 2005; Kim et al., 2009). Moreover, miRNAs and their target genes constitute complicated regulatory networks that contribute to the fine-tuning of various biological processes, such as cell proliferation, differentiation, and apoptosis (Miska, 2005; Hwang and Mendell, 2006). It has been demonstrated that miRNAs manipulate more than 30% of human genes, governing all cellular, physiological, and developmental processes. The majority of miRNAs exist intracellularly, while some exist in body fluids, including a variety of extracellular biologic fluids, such as blood, urine, saliva, pancreatic juice, and breast milk (Weber et al., 2010). Under physiological and pathophysiological conditions, exosomes are released through two slightly different mechanisms of “unconventional” exocytosis into the extracellular milieu by several cell types. Whether the packing of miRNAs into exosomes takes place at the pre- or mature-miRNA level has not yet been fully clarified. It has been demonstrated that the sequence motifs present in mature miRNAs can control their sorting into exosomes, and the ubiquitous hnRNPA2B1 recognizing these motifs binds exosomal miRNAs and specifically controls their loading into exosomes (Villarroya-Beltri et al., 2013).

miRNAs dysregulation associated with cancer progression, is common in all human cancers, including OSCC (Lee et al., 2008; Tahiri et al., 2014). Therefore, miRNAs have the potential to be used for the diagnosis and treatment of OSCC (Xu et al., 2016; Božinović et al., 2019; Kirave et al., 2020). miRNAs can be packed into microparticles and exosomes, resulting in transfer of miRNAs to body fluids by a passive release mechanism (Chen et al., 2008; Chim et al., 2008). Exosomes can also be released by tumor cells or circulating microvesicles via shedding as an active secretion mechanism (Shah and Calin, 2013). Exosomal miRNAs from the TME exert diverse effects on tumorigenesis, including the regulation of host immune responses, tumor growth, angiogenesis, metastasis, tumor chemoresistance, and control of the TME. Exosomal miRNAs derived from tumor cells affect the immune activity of several tumor-associated immune cells as well as the transport of signaling molecules among tumor cells, immune cells, and other cell types.

### Exosomal miRNAs and OSCC Progression

The contemporary therapies for OSCC yet are inefficient due to the limited understanding of their underlying mechanisms and the difficulties posed for developing accurate diagnostic methods. As important genetic materials transported in exosomes, exosomal miRNAs could serve as potential biomarkers and therapeutic targets for the treatment of OSCC. MiR-24-3p from salivary exosomes has been reported as a potential biomarker for OSCC (He et al., 2020). Furthermore, exosomal miRNAs play an essential role in growth, metastasis, and drug resistance. Exosomal miR-382-5p and miR-34a-5p from CAFs influence the proliferation, migration, and invasion of OSCC (Li et al., 2018; Sun et al., 2019). Li et al. (2018) showed miR-34a targeting AXL through AKT/GSK-3β/β-catenin pathway to promote the OSCC progression. Liu et al. (2017) first acquired cisplatin-resistant OSCC cells and used the conditional medium from resistant cells to treat parent OSCC cells. They further revealed that cisplatin-resistant OSCC cells could transfer miR-21 by exosomes targeting PTEN and PDCD4 to confer the cisplatin-resistance of the parental OSCC cells (Liu et al., 2017). Thus, exosomes may have the function as a vector for resistance transfer in cancer cells, and the resistance-related factors should be considered as therapeutic targets for effective treatment of OSCC.

Differential miRNA contents in OSCC exosomes have been reported in both pre-clinical and clinical studies. Recently, Rabinowits et al. (2017) compared the miRNA content of OSCC-derived exosomes with matching benign tissue and plasma from a patient. They found seven downregulated and nine upregulated miRNAs in tumor tissue compared with adjacent tissues. Furthermore, OSCC cells secreted miR-24-3p, miR-891a, miR-106a-5p, miR-2a-5p, and miR-1908 decreases the T-cell response in the tumor stroma by targeting the Mark1 signaling pathway and subsequently manipulating the proliferation and differentiation of cells (Ye et al., 2014). Moreover, miR-142-3p derived from exosomes were found to reduce TGFBR1 activity and promote OSCC cell proliferation *in vitro* and *in vivo* (Dickman et al., 2017). Exosomal miR-29a-3p derived from OSCC cells enhances tumor growth in a nude mouse model and M2 macrophage polarization by targeting the SOCS1 (Cai et al., 2019). Tachibana et al. (2016) and Xie et al. (2019a) showed that miR-223 and miR-101-3p function as tumor suppressors by inhibiting cell proliferation and inducing apoptosis through the process of exosome secretion, and exosomes secreting miR-338 from OSCC cells were also identified as tumor suppressors. Moreover, it was demonstrated that the overexpression of miR-34a-5p suppresses the proliferation of both CAL-27 and SCC-15 cells (Rabinowits et al., 2017). In addition, based on the colony formation assay, exosomal miR-34a-5p overexpression significantly reduced the colony counts of both CAL-27 and SCC-15 cells (Li et al., 2018).

Increased miRNA expression in exosomes is believed to promote OSCC metastasis. Leukoplakia is a precancerous lesion in OSCC, and it was found that miR-21 secreted from OSCC cells was correlated with low expression of its target genes, TPM1 and PTEN, and was highly expressed in progressive leukoplakia and OSCC to promote disease progression (Liu et al., 2017). Similarly, the involvement of exosome-delivered miRNAs in OSCC metastasis has been reported. Further analysis of six selected miRNAs revealed that miR-200c-3p silences its targets, CHD9 and WRN, as a key exosomal miRNA to promote tumor invasion that significantly accelerates the invasive potential of OSCC cells (Kawakubo-Yasukochi et al., 2018). OSCC-derived exosomes may influence cell motility and angiogenesis that, in turn, can influence OSCC progression. Two oncogenic miRNAs, miR-342-3p and miR-1246, are highly expressed in OSCC exosomes, leading to the metastasis of OSCC and increasing cell motility and invasive ability. miR-1246 directly targets DENND2D to promote the motility of tumor cells (Sakha et al., 2016). Thus, miRNAs in exosomes may be considered as non-invasive biomarkers for OSCC screening. On the contrary, inhibitory miRNAs may be delivered with exosomes to treat OSCC.

### Exosomal miRNAs and the OSCC Microenvironment

The TME contains a complex network of non-malignant cells, molecules, structural components, and chemicals that surround cancer cells. Multiple non-malignant cells, including endothelial cells, pericytes, immune cells, and fibroblasts, together with the surrounding ECM, comprise the supportive stroma of the tumor and manipulate the TME. The “seed and soil” hypothesis is widely accepted in the cancer field (Paget, 1989; Fidler and Poste, 2008). The pre-metastatic niche, conceptualized as a fertile soil conducive to the survival and growth of metastatic seeds, consists of diverse cell populations, such as CAFs and various infiltrating immune cells, and non-cell components of the ECM. These niche components influence the fate of disseminated tumor cells in diverse ways, such as cell proliferation and differentiation, and contribute to tumor angiogenesis, invasion, and metastasis (Wu et al., 2018; Peltanova et al., 2019). Exosomes have been identified as a crucial means of cell-to-cell communication, involving both near and distant signal transduction. Thus, tumor-derived exosomes can serve as messengers in the tumor environment, creating favorable environment for tumor growth and metastasis (Bae et al., 2018).

Alteration of the TME is the first step in forming a pre-metastatic niche. As one of the most abundant constituents of the TME, we demonstrated that CAFs perform critical roles during tumor progression and metastasis (Vu et al., 2019). miRNAs from cancer-derived exosomes are crucial messengers in the intercommunication between tumor cells and CAFs within the TME. Bovy et al. (2015) demonstrated that exosomes derived from CAFs enhance OSCC cell metastasis. Besides, fibroblasts in the TME “communicate” with tumor cells through the transfer of miRNAs contained in exosomes (Bovy et al., 2015). Li et al. (2018) found that the expression of miR-34a-5p in CAF-derived exosomes was significant, thereby inducing the EMT and reducing expression of the cancer stem marker AXL to facilitate cancer cell metastasis via the AKT-GSK3β-β-catenin signaling pathway. Furthermore, the miR-34a-5p-AXL axis enhanced nuclear translocation of β-catenin, thereby inducing transcriptional upregulation of SNAIL, which in turn activated the ECM proteins MMP-2 and MMP-9. Besides, it was found that miR-3188 expression by directly targeting to BCL2, is reduced in exosomes and their parental CAFs from OSCC tissues (Wang et al., 2019b). Sun et al. (2019) discovered that exosomal miR-382-5p derived from CAFs and NFs upregulates MMP-3, MMP-9, N-cadherin, and β-catenin in OSCC cells, thus increasing the migration of CAL-27.

The composition and function of the vasculature in the TME exhibits abnormalities, including leakiness, a heterogeneous basement membrane, irregular vessel branching, and poor pericyte coverage. These changes ultimately lead to a hypoxic TME (Hu and Polyak, 2008), and TEX can then be induced to migrate and invade normoxic cells. Li et al. (2016) showed that a hypoxic microenvironment may stimulate OSCC tumors to produce miR-21-rich exosomes, enabling miR-21 to be transported to normoxic regions and drive non-hypoxic cells toward a pro-metastatic phenotype.

The human immune system exerts its defensive functions by innate immunity and adaptive immunity. Innate immunity provides the body with its instinctive defense against the pathogenic infections, while their propagation brings about activation of adaptive immune responses. The infiltration of lymphocytes, including regulatory T cells (Tregs), MDSCs, and tumor-associated macrophages, is common in OSCC. By dampening the immune response and generating immune tolerance, these lymphocytic cells promote immune evasion by tumor cells. Li et al. (2019b) suggested that exosomal miR-21 plays a key role in the regulation of TEX-induced γδ T-cell function by affecting MDSCs. These authors utilized lenti-miR-138 virus γδ T cell-derived exosomes (γδTDEs) as a drug delivery system in the treatment of OSCC (Xie et al., 2019a). Delivery of miR-138 with γδTDEs had a synergistic inhibitory effect on CAL-27 cells *in vitro* (Xie et al., 2019a). In immunocompetent C3H mice, applying miR-138-rich γδTDE as a form of pre-immunization inhibited the growth of OSCC (Xie et al., 2019a). Using differential fluorescence, miR-101-3p was found to be transferred from donor hBMSCs to recipient TCA8113 cells. By targeting COL10A, the transferred miR-101-3p significantly repressed cell invasion and migration (Li et al., 2019c). Further evidence showed that the miR-21-5p that was released from CAL27-derived exosomes was taken up by THP1 monocytes, playing a role in activating the NF-κB inflammatory pathway. The delivery of miR-21-5p by exosomes promotes monocyte migration and infiltration, which in turn participates in the promotion of angiogenesis in OSCC (Momen-Heravi and Bala, 2018).

Interestingly, alcohol treatment (25 mM for 24 h) increases exosome production and alters the subset of oncogenic miRNAs that are specifically enriched in exosomes released from tumor cells (Momen-Heravi and Bala, 2018). Mechanistically, exosome uptake from OSCC cells by monocytes causes activation of the NF-κB pathway and establishment of a pro-inflammatory milieu (Momen-Heravi and Bala, 2018). Thus, TEXs promote changes in the microenvironment, such as oxygen reduction and decreased immune responses, this deterioration of the microenvironment exacerbates the progression of OSCC. However, the presence of exosomes from immune cells in the OSCC microenvironment was not reported.

**FIGURE 2:**
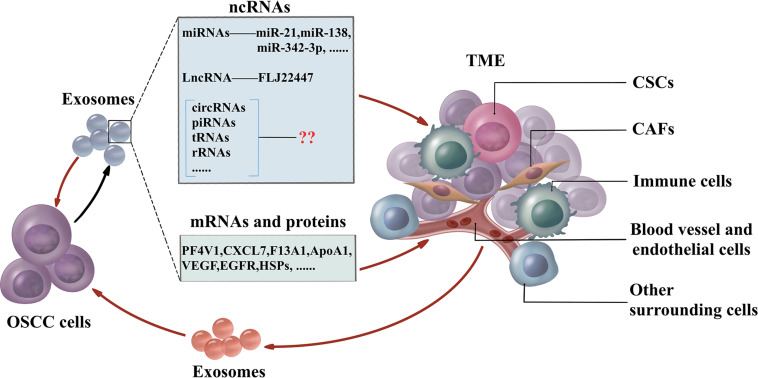
The feedback regulation between OSCC and TME. OSCC cells generate exosomes which include non-coding RNAs (miRNAs, lncRNAs, circRNAs, piRNAs, tsRNAs, and rRNAs), mRNAs, and proteins to influence the TME. CAFs, CSCs, immune cells, blood vessels, endothelial cells, and other surrounding cells are the essential cells in TME. Furthermore, cells in TME influence the function of OSCC cells by exosomes. OSCC cells also interact with adjacent OSCC cells via exosomes. OSCC, oral squamous cell carcinoma; TME, tumor microenvironment; miRNAs, microRNAs; lncRNAs, long non-coding RNAs; circRNAs, circular RNAs; piRNAs, PIWI-interacting RNAs; tRNAs, transfer RNAs; rRNAs, ribosomal RNAs; CAFs, cancer-associated fibroblasts; CSCs, cancer stem cells.

The traditional therapies for treating OSCC are surgery followed by chemotherapy. Due to the complexity of the TME, conventional drug delivery systems fail to transfer chemotherapeutics in an effective concentration to kill cancer cells and are associated with debilitating side effects. Exosomes have the essential characteristics including biocompatibility, non-cytotoxicity, low immunogenicity, simple to produce and store, long life span, and high cargo loading capacity (Steinbichler et al., 2019). The small size confers exosomes resistant to lung clearance and passing through the blood–brain barrier effectively (Kawikova and Askenase, 2015). Furthermore, exosomes may be used as specific targeting against cancer cells rather than normal cells by receptors in exosomes (Wang et al., 2016). Mounting evidence has provided insights about the crucial role of exosomal miRNAs in controlling the TME, and these insights could be applied to drug delivery. These miRNAs could be utilized as therapeutic components delivered to the OSCC microenvironment by exosomes. Exosome-delivered tumor suppressor miRNAs, miR-143 inhibits the growth of prostate cancer, while the let-7a significantly reduces the growth of breast cancer *in vivo*, respectively (Kosaka et al., 2012; Ohno et al., 2013b). Bio-safety has also been confirmed by adverse effects detection in normal prostatic epithelial cells with treatment of exosome-containing miR-143 (Kosaka et al., 2012). Cancer may acquire the drug resistance against chemotherapeutics by drug efflux with transporters. Exosomes could deliver the anti-miR-9 to reduce the transporter level, thus, sensitizes glioblastoma cells to temzolomid to increase cell death (Munoz et al., 2013). The feedback regulation of exosomes and TMEs is shown in Figure 2.

## Exosomal lncRNAs, Circular RNAs, and piRNAs in OSCC

Aside from miRNAs, ncRNAs also consist of long non-coding RNAs (lncRNAs), circular RNAs (circRNAs), and piRNAs, rRNAs and tsRNAs. lncRNAs are defined as transcriptional products, with a length of more than 200 nucleotides and generally having no protein-coding potential (Jin et al., 2016). However, ncRNAs involve in multiple pathological processes. Gastrointestinal cancer has the same problem as OSCC of resistance to chemotherapy agents, such as cisplatin.

### Exosomal lncRNAs

Exosomal lncRNAs mediates the progression and chemoresistance of tumor cells in the TME through diverse mechanisms. The exosomal lncRNA HOTTIP, transmitted from cisplatin-resistant gastric cancer cells to sensitive cancer cells, plays a role in conferring cisplatin resistance to sensitive cancer cells by binding to miR-218 to activate HMGA1 (Wang et al., 2019a). Moreover, this lncRNA is upregulated in the bodily fluids of gastric cancer patients, which indicates that it is a potential biomarker for early diagnosis and treatment. Similarly, Kogure et al. (2013) reported that the exosomal lncRNA TUC339 secreted by hepatocellular cancer cells is absorbed by surrounding cells and thereby promotes the growth of hepatocellular carcinoma. Ding et al. (2018) found that the lncRNA FLJ22447 derived from OSCC exosomes is upregulated in CAFs and activates them to induce the proliferation of OSCC cells through IL-33.

### Exosomal CircRNAs

CircRNAs are a kind of single-stranded RNA that is comprised of mostly cytoplasmic exonic particles with linked 3′ and 5′ ends in eukaryotic cells. Several circRNAs have been found in body fluids, such as blood and saliva (Holdt et al., 2018; de Fraipont et al., 2019). circRNAs sorting into exosomes may be controlled by modulation of associated miRNA levels in parental cells and may transfer biological activity to target cells (Li et al., 2020). A recent study has reported that the exosomal circRNAs DB, derived from adipocytes, promotes the growth of hepatocellular carcinoma by sponging miR-34a and activating USP7/Cyclin A2 signaling pathway (Zhang et al., 2019). Zhao et al. (2019) found that the circRNA ATP8B4 acts as miR-766 sponge and plays a role in the development of radiation resistance in glioblastoma. The circRNA CDR1as functions as a miR-7 sponge, regulating insulin transcription and secretion in pancreatic islet cells via miR-7 targets and downregulates miR-7 to perturb the development of diabetes (Xu et al., 2015). Hansen et al. (2013) introduced miR-7 mimics into HEK293T and MCF-7 cell lines and reported that the level of exosomal CDR1as is significantly decreased while increased in cells due to the ectopic expression of miR-7 in both HEK293T and MCF-7 cells.

### Exosomal piRNAs

piRNAs are abundant small, non-coding, single-stranded RNAs 21-30 nucleotides in length, with little conservation of sequence between organisms (Weick and Miska, 2014). Compared with miRNAs, piRNAs function to repress transposons at the transcriptional and posttranscriptional levels to maintain genome integrity, while miRNAs play a role in repressing translation at the post-transcriptional level to regulate gene expression (Iwasaki et al., 2015). Although piRNAs have only 1–4% of all identified sequence content in the exosome, it was found that piRNAs are as abundant as miRNAs in exosomes isolated from plasma (Yuan et al., 2016). piRNAs are regarded as potential biomarkers in breast cancer, colon, and gastric cancer for pathological expression (Cheng et al., 2011; Hashim et al., 2014; Vychytilova-Faltejskova et al., 2018). Mounting evidence shows that piRNAs strongly correlate with tumor cell malignant phenotype and clinical stage. piR-021285 regulates cell proliferation and invasion by DNA methylation. The piR-021285 variant mimics transfection into breast cancer cell lines and weakens pro-invasive and pro-apoptosis gene methylation of ARHGAP11A at the 5′-UTR-first exon CpG site, which results in higher ARHGAP11A expression and increased breast cancer cell invasiveness (Fu et al., 2015). piRNAs are also involved in the development of lung cancer. The tumor promoter RASSF1C upregulates piR-34871 and piR-52200 and downregulates piR-35127 and piR-46545 through the RASSF1C-PIWIL1-piRNA axis, resulting in the promotion of stem cell proliferation and the EMT in lung cancer. These piRNAs changes inhibit AMPK phosphorylation in the ATM-AMPK-p53-p21cip pathway and thereby block cell cycle arrest and enhance cell proliferation (Reeves et al., 2012). Moreover, we developed chemical induced OSCC mouse model and found some piRNAs were significantly changed. And piRNAs has been identified in the exosomes of mesenchymal stem cells (Wang et al., 2020). However, the exosomal function of piRNAs in human OSCC should be further revealed.

Generally, exosomal lncRNAs, circRNAs, and piRNAs are involved in the tumor development. However, there are few reports on the involvement of these exosomal ncRNAs in the functioning of OSCC and their potential as biomarkers in OSCC, which deserves more attention and deeper exploration.

## mRNAs and Proteins in OSCC

Proteins contained inside exosomes have also been evaluated in patients with OSCC, although not as extensively as miRNAs, and studies have shown promising exosomal protein markers for early diagnosis of OSCC, such as TRAP1, EGFR, heat shock protein 90 (HSP-90), and MMP-13, which can affect the intracellular functions of genes (Kaskas et al., 2014; Xie et al., 2019b). Among the proteins contained in exosomes, 23 were identified as potential biomarkers of OSCC (Boldrup et al., 2017). Recent studies found that the contents of free exosomes in blood were correlated with OSCC cells. Proteins in those exosomes, including PF4V1, CXCL7, F13A1, and ApoA1, could be used in the diagnosis of OSCC (Li et al., 2019a). Angiogenesis is generally correlated with tumor growth and metastasis, and exosomes derived from OSCC cells could have an inhibitory or promotional effect on angiogenesis, thereby influencing OSCC metastasis (Zhang et al., 2019). Rosenberger et al. (2019) demonstrated that exosomes manipulate the secretion of VEGF to inhibit the angiogenic activity of endothelial cells, thus reducing tumor metastasis. It was found that exosome treatment inhibits angiogenic activity, including both vessel density and vascular area.

The EMT also plays an essential role in tumor migration and invasion. Overexpression of EGFR is an essential feature of OSCC. It was found that OSCC cells abundantly express EGFR, which is secreted from cells as OSCC exosomes upon EGF stimulation (Fujiwara et al., 2018). Furthermore, OSCC LNM was always found in patients who were diagnosed in the later stages of the disease. Li et al. (2019a) isolated exosomes from the serum of OSCC patients, and found that the exosomal proteins PF4V1, CXCL7, F13A1, and ApoA1 in serum affect OSCC LNM and thereby influence prognosis. ROC analysis is a kind quantification method to acquire desirable levels of sensitivity and specificity (Obuchowski and Bullen, 2018). ROC analysis using the relative abundances of ApoA1, CXCL7, PF4V1, and F13A1 in serum, serum exosomes, and whole blood indicated that exosomal proteins are potentially predictive biomarkers for OSCC with LNM.

Cancer cells often secrete exosomes carrying heat shock proteins, which play a part in tumor progression. It was found that abundant secretion of exosomes rich in HSP-90 was found in OSCC with LNM, indicating a poor prognosis (Ono et al., 2018). Besides, drug resistance also remains a severe problem in most chemotherapy treatments for OSCC (Samuel et al., 2017). Recently, it has been suggested that cancer cell-derived exosomes mediate drug resistance. Khoo et al. (2019) showed the increased exosome production in both *de novo* (H314) and adaptive (H103/cisD2) resistant cell lines compared with sensitive H103 cells. Moreover, differences in the proteomes contained within exosomes indicate that adaptation to cisplatin treatment causes significant changes in the secreted exosomes (Khoo et al., 2019). The cargos in the exosomes were showed in Table 1.

**TABLE 1:** Cargos in exosomes derived from OSCC.

Biomolecules	Function	Molecules	Exosome origin	References
miRNAs	Suppressor	miR-338, miR-24-3p, miR-891a, miR-106a-5p, miR-2a-5p, miR-1908, miR-101-3p	Cell lines	Ye et al., 2014; Tachibana et al., 2016; Xie et al., 2019a
	Biomarker	miR-223	Plasma	Tachibana et al., 2016
	Metastasis	miR-21, miR-142-3p, miR-29a-3p, miR-342-3p, miR-1246, miR-200c-3p	Cell lines	Sakha et al., 2016; Dickman et al., 2017; Liu et al., 2017; Kawakubo-Yasukochi et al., 2018; Cai et al., 2019
	Accumulation of fibronectin	miR-382-5p, miR-34a-5p, miR-3188	CAFs	Bovy et al., 2015; Liu et al., 2017; Li et al., 2018
	Motility	miR-342-3p, miR-1246,	Cell lines	Sakha et al., 2016
	Angiogenesis	miR-21	Cell lines	Momen-Heravi and Bala, 2018
	Immune regulation	miR-138,	Cell lines	Xie et al., 2019a
Proteins	Biomarker	PF4V1, CXCL7, F13A1, ApoA1	Serum	Li et al., 2019a
		VEGF, EGFR, HSPs	OSCC cells	Fujiwara et al., 2018; Ono et al., 2018; Rosenberger et al., 2019
lncRNAs	Proliferation	Lnc-FLJ22447	OSCC cells	Ding et al., 2018

*CAFs, cancer-associated fibroblasts; HSPs, heat shock proteins; EGFR, epidermal growth factor receptor; VEGF, vascular endothelial growth factor; PF4V1, platelet factor 4 variant 1; CXCL7, C-X-C motif chemokine ligand 7; F13A1, coagulation factor XIII A chain; ApoA1, apolipoprotein A1.*

## Discussion

In 2012, miRNAs were found in the exosomes of invasive tumors, suggesting that tumor-derived exosomes may serve as an important diagnostic tool to avoid metastasis and improve prognosis (Chen et al., 2012a). Therefore, it is essential to determine the involvement of exosomes in maintaining the aggressive phenotype of OSCC cells and their unique roles in intercellular communication.

Although several miRNAs have been identified in the exosomes of OSCC, more should be analyzed by improving the isolation and purification of exosomes. Furthermore, the function of lncRNAs, circRNAs, piRNAs, mRNAs, and proteins in the exosomes of OSCC also needs to be determined. In fact, tRNAs and rRNAs are infrastructural ncRNAs, they are also involved into the cancer initiation and progression. tRNAs re small conserved RNA molecules that allow the translation of the genetic code into amino acids. Overexpression of initiator tRNA^Met^ (tRNA${}_{\text{i}}^{\text{Met}}$) is to promote metastasis of melanoma cells, through α5β1 integrin-dependent signaling (Birch et al., 2016). Furthermore, the levels of tRNA^Glu^UUC and tRNA^Arg^CCG are increased in metastatic breast cancer cell lines (Goodarzi et al., 2016). Dysregulated rRNA transcription is essential in cancers and may be involved in the initiation stage of hepatocarcinogenesis (Donati et al., 2011; Xie et al., 2018). However, whether rRNAs are existed in exosomes and the exosomal rRNAs should be further revealed.

**FIGURE 3:**
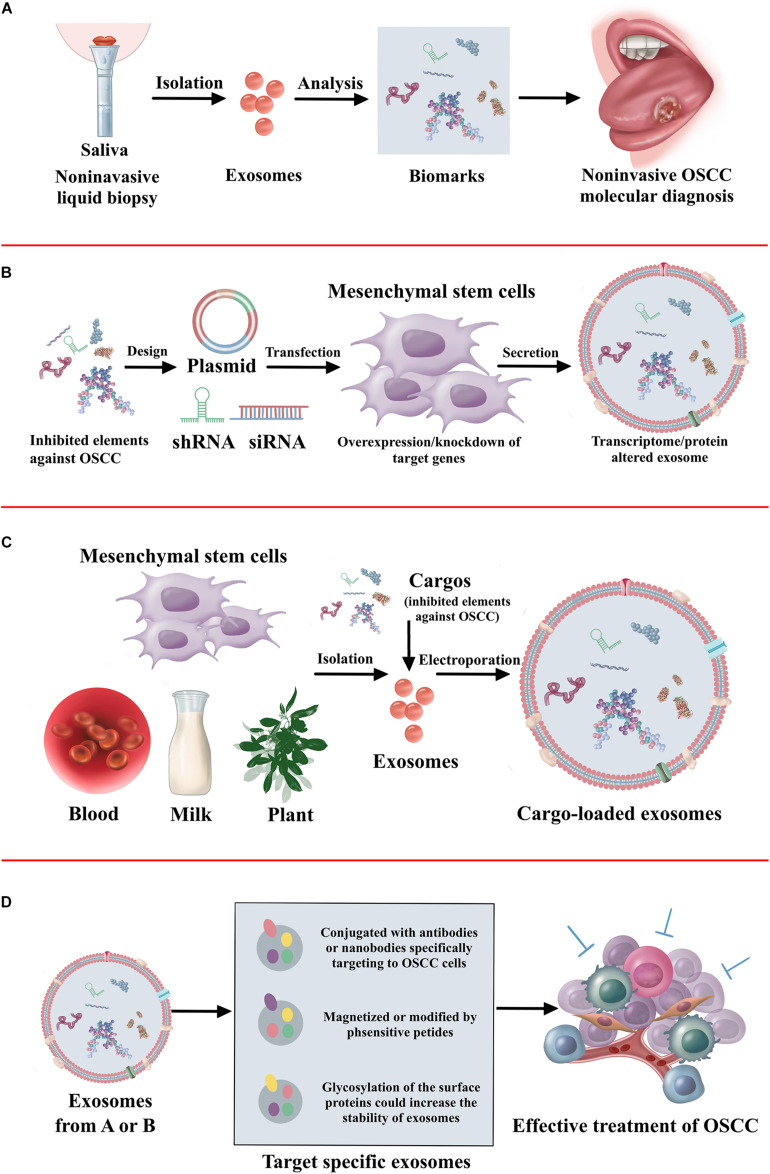
The summary of therapeutic application of exosomes. **(A)** The non-invasive diagnosis and prognosis of OSCC using exosomes. **(B)** Approaches to alter cargo contents in the mesenchymal stem cell-derived exosomes to treat OSCC. **(C)** Loading of exogenous proteins, genes, or signaling molecules in the exosomes to treat OSCC. **(D)** Exosome surface modification for target specific delivery of exosomes toward OSCC.

As a viable alternative to tissue-based sampling in the clinic, it is clear that there is a great deal of interest in non-invasive liquid biopsies (Xue et al., 2019). Liquid biopsies utilize blood and saliva to detect the circulating tumor cells, circulating tumor DNA, and exosomes for the diagnosis and prognosis of oral cancer (Lousada-Fernandez et al., 2018). This method allows repeated sampling to monitor the treatment response, assess tumor heterogeneity, and even use in cancer screening programs. Wu et al. (2017) has shown that an acoustofluidic platform integrating acoustics and microfluidics efficiently isolates exosomes directly from undiluted blood samples and saliva. Saliva collection was rather easy and non-invasive. It has demonstrated that exosomes have a higher amount and larger size in the saliva of patients with oral cancer than healthy individuals (Zlotogorski-Hurvitz et al., 2016; Nair et al., 2018). Furthermore, increased expression of CD63 and lesser expression of CD9 and CD81 are found in the saliva exosomes of oral cancer patients (Zlotogorski-Hurvitz et al., 2016). More efforts should be made to identify new biomarkers in the exosomes of saliva, finally increasing the application in non-invasive cancer diagnosis. Considering the endogenous transport function, exosomes have robust potential to be applied as therapeutic delivery systems. However, efficiency in delivering drugs to the tumor is still a major challenge due to the blood–brain barrier and degradation (Kawikova and Askenase, 2015). Exosomes can be fused with the cell membrane with high reliability, and therefore non-coding RNAs or peptide drugs may be packed into exosomes and delivered to the OSCC tumor. Furthermore, the process of exosomes transfected with ncRNAs may influence the activity of exosomes. Thus, ncRNAs could be transfected into the exosome derived cells. It has demonstrated exosomes from mesenchymal stem cells overexpressed with tumor suppressed miRNAs has inhibitory effect on tumor progression (Che et al., 2019; Xu et al., 2019; Yuan et al., 2019). Furthermore, the fabrication of target-specific exosomes could increase the efficacy of cancer treatment (Wang et al., 2016). The exosomes conjugated with antibodies specifically target cancer cells (Stickney et al., 2016). The exosomes magnetized or modified by pH-sensitive peptide also contributes to the accumulation in the cancer cells (Nakase and Futaki, 2015; Qi et al., 2016). Furthermore, the glycosylation of the surface proteins increases the stability of exosomes (Hung and Leonard, 2015). The therapeutic application of exosomes against OSCC is illustrated in Figure 3.

In summary, it is essential to understand the role of exosomes in influencing tumor phenotype, angiogenesis, immune modulation, metastasis, and drug resistance. While studies on the role of exosomes in OSCC have made progress, there are several outstanding questions that need to be further explored. The versatile biological functions of exosomes could be promising tools to apply in the diagnosis, prognosis, and effective treatment of OSCC.

## Author Contributions

LWu, MZ, and WC concepted the manuscript. YL, ZZ, and YY wrote the original draft. JP, XY, LWa, LWu, and WC revised the manuscript. ZY made the improvement for the figures. All authors contributed to the article and approved the submitted version.

## Conflict of Interest

The authors declare that the research was conducted in the absence of any commercial or financial relationships that could be construed as a potential conflict of interest.

## Abbreviations

OSCC: oral squamous cell carcinoma
EVs: extracellular vesicles
MVB: multivesicular body
CHB: chronic hepatitis B
TME: tumor microenvironment
ESCRT: endosomal tethering complexes necessitated for transport
GTPase: Rab guanosine triphosphatase
PEG: Polyethylene glycol
ncRNAs: non-coding RNAs
miRNAs: microRNAs
hnRNPA2B1: heterogeneous nuclear ribonucleoprotein A2B1
CAFs: cancer-associated fibroblasts
PTEN: phosphatase and tensin homolog
PDCD4: programmed cell death 4
Mark1: microtubule-affinity-regulating kinase 1
TGFBR1: type I TGF-beta receptor
SOCS1: suppressor of cytokine signaling 1
TPM1: tropomyosin alpha-1 chain
CHD9: chromodomain helicase DNA binding protein 9
WRN: Werner syndrome gene
DENND2D: DENN/MADD domain containing 2D
ECM: extracellular matrix
EMT: epithelial-mesenchymal transition
MMPs: extracellular matrix proteins
NFs: normal fibroblasts
BCL2: B-cell lymphoma 2
Tregs: regulatory T cells
MDSCs: myeloid-derived suppressor cells
TEX: hypoxic tumor-derived exosome
lncRNAs: long non-coding RNAs
circRNAs: circular RNAs
piRNAs: P-element -induced wimpy testis (PIWI)-interacting RNAs
rRNAs: ribosomal RNAs
tsRNAs: transfer RNAs
HMGA1: the high mobility group A1
USP7: ubiquitin-specific protease-7
ARHGAP11A: Rho GTPase activating protein 11A
ATMs: ataxia telangiectasia mutated
AMPK: adenosine monophosphate-activated protein kinase
TRAP1: Tumor necrosis factor receptor-associated protein 1
EGFR: epidermal growth factor receptor
HSP: heat shock protein
PF4V1: platelet factor 4 variant 1
CXCL7: C-X -C motif chemokine ligand 7
F13A1: Coagulation factor XIII A chain
ApoA1: apolipoprotein A1
VEGF: vascular endothelial growth factor
LNM: lymph node metastasis
ROC: receiver operating characteristic.
